# LncRNA GAS6‐AS2 promotes bladder cancer proliferation and metastasis via GAS6‐AS2/miR‐298/CDK9 axis

**DOI:** 10.1111/jcmm.13986

**Published:** 2018-11-05

**Authors:** Xin Rui, Li Wang, Huafeng Pan, Tingting Gu, Siliang Shao, Jiangyong Leng

**Affiliations:** ^1^ Department of Urology Ningbo No. 2 Hospital Ningbo China

**Keywords:** bladder cancer, GAS6‐AS2, metastasis, proliferation

## Abstract

Long noncoding RNAs (lncRNAs) have been proved to play important roles in carcinogenesis and development of numerous cancers, but their biological functions in bladder cancer remain largely unknown. In this study, a novel lncRNA termed GAS6‐AS2 were primary identified, and its roles as well as mechanisms in regulating proliferation and metastasis of bladder cancer cells were investigated. Clinically, GAS6‐AS2 was significantly up‐regulated in bladder cancer tissues and positively correlated with tumour stages and poor prognosis. Moreover, expression of GAS6‐AS2 was also increased in bladder cancer cells compared with normal bladder cells. Further investigating the roles of GAS6‐AS2, we found GAS6‐AS2 regulated proliferation and proliferative activity of bladder cancer cells via inducing G1 phase arrest. What's more, we found that GAS6‐AS2 contributed to metastatic abilities of cells. In mechanism, GAS6‐AS2 could function as a competitive endogenous RNA (ceRNA) via direct sponging miR‐298, which further regulating the expression of CDK9. Finally, we also proved that GAS6‐AS2 knockdown suppressed tumour growth and metastasis in vivo. In conclusion, our study proved that GAS6‐AS2 could function as a ceRNA and promote the proliferation and metastasis of bladder cancer cells, which provided a novel prognostic marker for bladder cancer patients in clinic.

## 1 INTRODUCTION

Bladder cancer (BC) is one of the most common malignant cancers of the urinary system in China, and its incidence and mortality rates have increased in recent years.^1^ It has been estimated that bladder cancer accounts for 38 600 new cases and causes ~15 000 mortalities worldwide annually.^2^ Up to now, little was known about the mechanisms of bladder cancers tumourigenesis and development, thus lacking of sensitive prognostic biomarker, it is of great significance to explore the mechanisms of BC and further providing prognostic biomarkers for clinical diagnosis and treatment of BC patients.^3^

Long noncoding RNAs (LncRNAs) are a cluster of RNAs which >200 nucleotides in length but lack protein‐coding capacity, and play important roles in numerous biological processes.^4^ Specifically, previous studies have showed that lncRNAs are dysregulated in cancers, which have been proved to regulate the carcinogenesis and progression via X chromosome inactivation, splicing, imprinting, epigenetic control, gene transcription regulation, and sponging microRNAs.^5^, ^6^, ^7^, ^8^, ^9^ Lots of lncRNAs have been identified in bladder cancer, including ANRIL, LINC00857, LSINCT5, and so on.^10^, ^11^ For instance, Dudek et al^11^ showed that linc00857 expression predicts and mediates the response to platinum‐based chemotherapy in muscle‐invasive bladder cancer. LSINCT5 activates Wnt/β‐catenin signalling by interacting with NCYM to promote bladder cancer progression.^12^ While the functions and mechanisms of lncRNAs are still remain largely unknown, it of great significance to explore novel lncRNAs and identify their functions, which might provide potential therapeutic targets for clinical treatments of bladder cancer patients.

In this study, we primarily identified a novel lncRNA termed GAS6‐AS2, and our research proved that GAS6‐AS2 contributed to proliferation and metastasis of bladder cancer cells via the GAS6‐AS2/miR‐298/CDK9 axis. The research broadens our insights into the underlying mechanisms in proliferation and metastasis, and provided a new therapeutic target for bladder cancer patients in clinic.

## 2 MATERIALS AND METHODS

### 2.1 TCGA bladder cancer and normal control sequencing data

TCGA database contains RNA sequencing data for multiple types of cancer. The RNA sequences of 19 normal control and 252 bladder cancer tissues were downloaded and analysed based on the Atlas of Noncoding RNAs in Cancer (TANRIC) database.^13^

### 2.2 Cell lines and cell culture

SV‐HUC‐1, RT4, 5637, and T24 cell lines (ATCC, Rockville, MD, USA) were cultured in Dulbecco's modified Eagle's medium supplemented with 10% foetal bovine serum (Aurogene) in a humidified atmosphere containing 5% CO_2_ at 37°C.

### 2.3 Construction of stable GAS6‐AS2 knockdown cell lines

GAS6‐AS2 specific shRNA vectors and its control was acquired from Vigene Biosciences (Rockville, MD, USA) and were transfected into T24 and 5637 cell lines using lipo Lipofectamine^®^ RNAiMAX Reagent (Thermo Fisher Scientific, Inc., Waltham, MA, USA) according to the manufacturer's protocol. The shRNA sequence to GAS6‐AS2 were sh1: 5′‐CTGTATGTACACTTTTTTGTC‐3′, sh2: 5′‐CTGGGAATGATCTTCAAGGAG‐3′.

### 2.4 Cell viability assay

Cell viability was determined by a 3‐(4, 5‐dimethylthiazol‐2‐yl)‐2, 5‐diphenyltetrazolium bromide (MTT, Sigma, Louis, MO, M2003) assay for 5 days. 20 μl of MTT (5 mg/mL in PBS) was added into each well and incubated for 4 hours. The supernatants were carefully aspirated, and 100 μl of dimethyl sulphoxide (DMSO) was added to each well. Absorbance values at 490 nm were measured on a Microplate Reader (Bio‐Rad, Hercules, CA, USA). Ethynyl deoxyuridine (Edu) assays was performed as previously described,^14^ and the cells were observed under a microscope and five fields were randomly selected to be photographed in 10× magnification.

### 2.5 Cell cycle assay

Cells were collected, washed twice with 1X PBS, and fixed in 70% ethanol at −20°C. After 24 hours of fixation, cells were incubated with RNase A (Takara Bio, Inc., Otsu, Japan) at 100 μg/mL in 1X PBS for 30 minutes at 37°C. Cells were then stained with propidium iodide (PI; BD Biosciences, San Jose, CA, USA) at 50 μg/mL for 30 minutes at room temperature. Subsequently, cells were analysed for DNA content using a BD FACSCalibur™ flow cytometer (BD Biosciences).

### 2.6 Wound healing assay

Cells were harvested and reseeded into 96‐well culture plates at the concentration of 3 × 10^4^/well and 2 × 10^4^/well, respectively. After 16 hours incubation, when cells had reached more than 90% convergence, the wound was performed, washed twice in PBS, replaced with serum‐free medium, and maintained in an incubator. Cells were photographed after 0 and 24 hours in 10× magnification, and the width of the wound was recorded as the wound distance.

### 2.7 Transwell assay

The cell migration and invasion abilities were analysed by a transwell assay, which was performed in 24‐well transwell chambers (Corning, NY, USA). After 24 hours transfection, cells in 200 μL serum‐free medium were seeded into the upper chamber (For invasion assay, the upper was cover with Matrigel). The lower chamber was filled with 600 μL medium with 20% FBS. After incubating, cells on the inner membrane were removed. The outer membrane was fixed with 4% paraformaldehyde and stained with 0.1% crystal violet solution. The cells were observed under a microscope and five fields were randomly selected to be photographed in 10× magnification.

### 2.8 Dua‐luciferase reporter assay

The dual‐luciferase miRNA target expression vector pmirGLO (Promega, Madison, WI, USA) was used to generate luciferase reporter constructs. Full length GAS6‐AS2 sequence with wild‐type (WT) and mutant type (Mut) microRNA binding site were obtained from Vigene Biosciences. Cells were seeded in 96‐well plates and cotransfected with wild‐type or mutated GAS6‐AS2 constructs and miR‐298 mimic. Luciferase activity was measured with the dual‐luciferase reporter assay system (Promega). Firefly luciferase activity was normalized against Renilla luciferase activity.

### 2.9 RNA immunoprecipitation (RIP) assay

RIP experiments were performed using the Magna RIP RNA‐Binding Protein Immunoprecipitation Kit (Millipore, Billerica, MA, USA) according to the manufacturer's instructions. Antibody for RIP assays of AGO2, or control IgG were from Millipore. The coprecipitated RNAs were detected by qRT‐PCR. The total RNAs were the input controls.

### 2.10 Quantitative RT–PCR analysis

Total RNA was extracted from cells using the TRIzol reagent (Invitrogen, Carlsbad, CA, USA). PrimeScript reverse transcriptase (RT) reagent kit (TaKaRa, Shiga, Japan) was used to synthesize cDNA from total RNA. MiRNA from 1 μg of total RNA was reverse transcribed using the Prime‐Script miRNA cDNA Synthesis Kit (TaKaRa). Real‐time PCR was performed on Applied Biosystems StepOne plus System, and results were analysed as previously described.^15^ The primers used in our study included: GAS6‐AS2 F: AAGGAGGACGCAATACC; GAS6‐AS2 R: ATCCTGGCTAACACGGT; GAPDH F: GTCTCCTCTGACTTCAACAGCG; GAPDH R: ACCACCCTGTTGCTGTAGCCAA; MiR‐298: AGCAGAAGCAGGGAGGTTCTCCCA; U6: GCGCGTCGTGAAGCGTTC.

### 2.11 Western blot analysis

Western blot analysis was performed as described previously.^16^ In brief, cells were harvested and lysed in lysis buffer containing protease inhibitors. Subsequently, 50 μg of total cellular protein from each sample were separated by 10% SDS‐PAGE and electro‐transferred onto polyvinylidene fluoride (PVDF) membrane using a semidry blotting apparatus (Bio‐Rad). The membranes were blocked with 5% nonfat milk at room temperature for 1 hour, and then incubated with primary antibodies overnight at 4°C. After incubation with the appropriate secondary antibodies, the protein bands were detected using the Prolighting HRP agent. Expression of β‐actin was used as a loading control. The antibody used in this study was obtained from Abcam.

### 2.12 In vivo proliferation and metastasis assay

Briefly, T24 cells (1 × 10^7^ cells) in 200 μL of PBS: Matrigel (1:3, v/v) were injected subcutaneously into left flank of 4‐6 weeks‐old BALB/c nu/nu male mice (five mice per group). Tumour growth rate was monitored by measuring tumour diameters every 5 days. Both maximum (L) and minimum (W) length of the tumour were measured using a slide caliper, and the tumour volume was calculated as ½LW2. When mice were killed, tumours were collected. To produce experimental lung metastasis, 5 × 10^5^ cells were injected into the lateral tail veins of 4‐6 weeks‐old BALB/c nu/nu male mice (five mice per group). After 3 weeks, all the mice were killed under anaesthesia. The lungs were collected and fixed in 10% formalin.

### 2.13 Statistical analysis

All data are presented as means ± SD from at least three independent experiments. The software SPSS V18.0 (Chicago, IL, USA) was used for statistical analysis. Statistical significance of differences between two groups was evaluated using Student's *t*‐test, and one‐way ANOVA was used to determine the significance of differences among multiple groups. Differences with *P* < 0.05 were considered statistically significant.

## 3 RESULTS

### 3.1 GAS6‐AS2 is up‐regulated in bladder cancer tissues and cell lines

**Figure 1:**
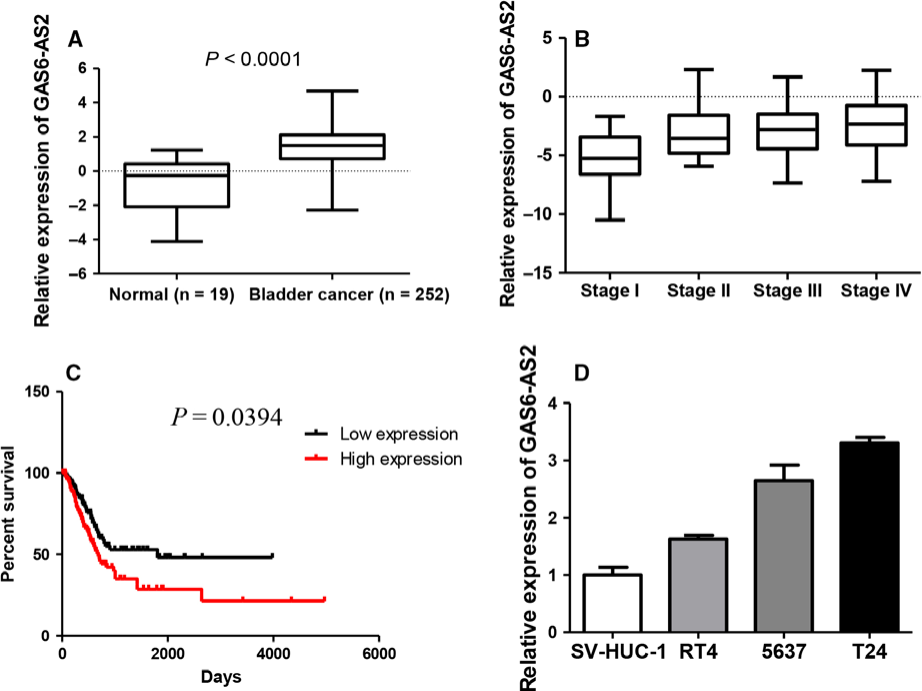
GAS6‐AS2 were overexpressed in bladder cancer tissues and cell lines and were correlated with prognosis. A, GAS6‐AS2 were overexpressed in bladder cancer tissues based on TCGA RNAseq database. B, GAS6‐AS2 expression was positively correlated with bladder cancer stages. C, GAS6‐AS2 overexpression predicted a poorer prognosis in bladder cancer patients. D, GAS6‐AS2 were overexpressed in bladder cancer cell lines. Data represent means ± SD of at least three independent experiments

To explore novel bladder cancer associated lncRNAs, we downloaded and analysed the RNA‐seq and clinical profiles from TCGA database. A total of 19 normal and 252 bladder cancer tissues were involved in our study, 218 lncRNAs were up‐regulated and 176 lncRNAs were down‐regulated in bladder cancer tissues. Among the most significantly changed lncRNAs, GAS6‐AS2 were up‐regulated in bladder cancer tissues (Figure 1A) and positively correlated with stages of bladder cancers (Figure 1B). What's more, we found GAS6‐AS2 overexpression indicated a poorer prognosis of bladder cancers (Figure 1C). Finally, we further evaluated the expression of GAS6‐AS2 in bladder cancer and normal cell lines and an up‐regulation in bladder cancer cell lines was found in accordance with above results.

### 3.2 Knockdown of GAS6‐AS2 suppresses bladder cancer proliferation via inducing G1 phase cell cycle arrest

**Figure 2:**
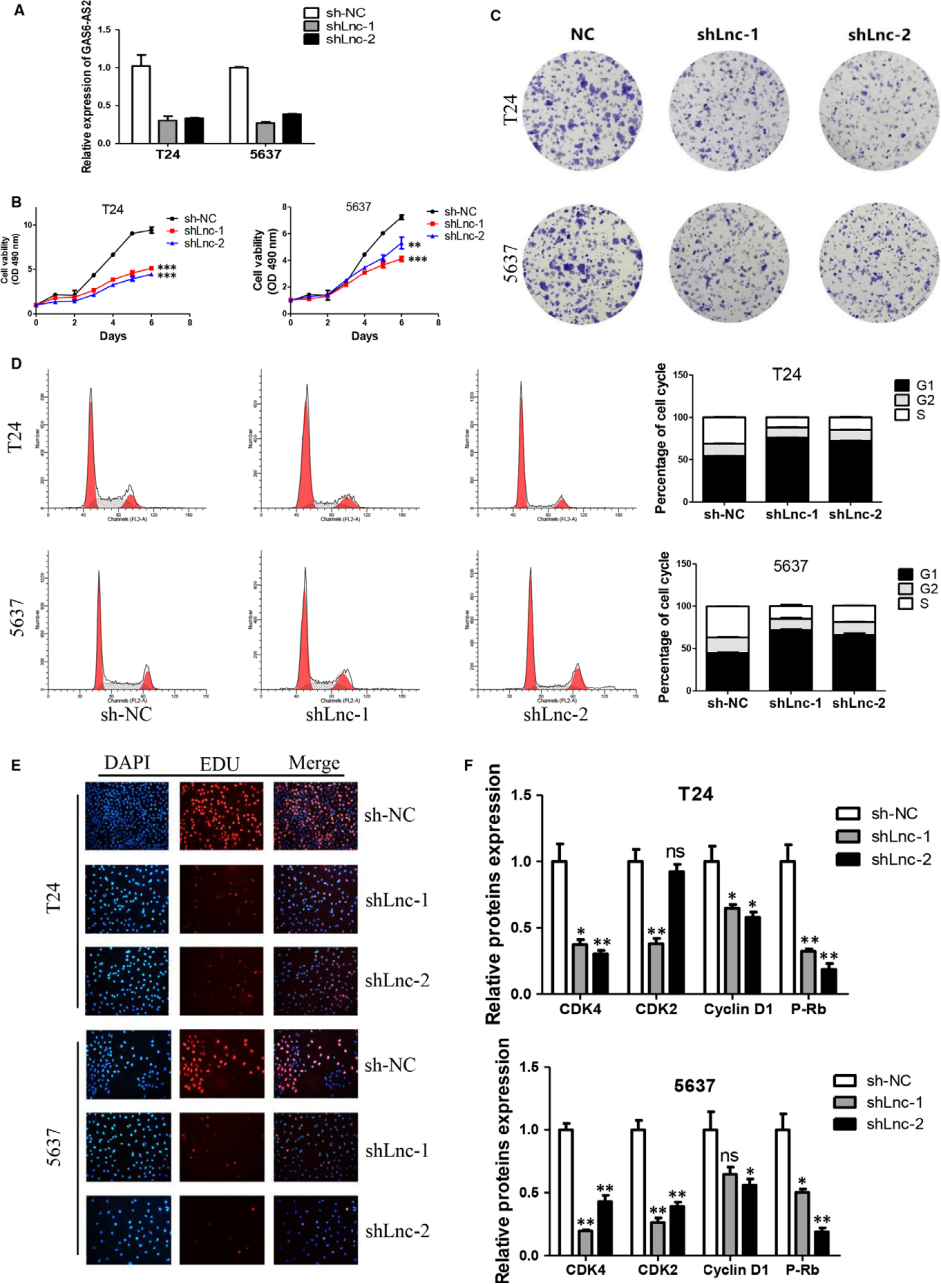
Knockdown of GAS6‐AS2 suppressed bladder cancer proliferation via induction of G1 phase arrest. A, GAS6‐AS2 were knockdown in both T24 and 5637 cells by two shRNA vectors. B, GAS6‐AS2 knockdown inhibited proliferation of T24 and 5637 cell lines. C, GAS6‐AS2 knockdown inhibited colony formation abilities of T24 and 5637 cell lines. D, GAS6‐AS2 knockdown contributed G1 phase cell cycle arrest in both cell lines. E, EDU assay confirmed reduced proliferation abilities after GAS6‐AS2 knockdown. F, GAS6‐AS2 knockdown altered cell cycle proteins. Data represent means ± SD of at least three independent experiments. *P < 0.05, **P < 0.01, ***P < 0.001

The effect of GAS6‐AS2 shRNA vector on RNA expression level was shown as Figure 2A. To evaluated roles of GAS6‐AS2 on cell proliferation, MTT and colony formation assay of T24 and 5637 cell lines were performed. As shown in Figure 2B and C, the proliferation ability of both cell lines was remarkable inhibited. As cell cycle arrest was one of the most important manners that contributes to proliferation suppression, we further examined the cell cycle after GAS6‐AS2 knockdown. We proved that knockdown of GAS6‐AS2 caused significant G1 phase cell cycle arrest (Figure 2D), and cell cycle checkpoint proteins were greatly inhibited (Figure 2E), which were in accordance with our results. Finally, Edu assay proved the proliferation abilities of both T24 and 5637 cell lines were significantly suppressed (Figure 2F). In conclusion, our results demonstrated knockdown of GAS6‐AS2 suppresses bladder cancer proliferation via inducing G1 phase cell cycle arrest.

### 3.3 Knockdown of GAS6‐AS2 inhibits metastatic abilities of bladder cancer cells via suppressing EMT pathways

**Figure 3:**
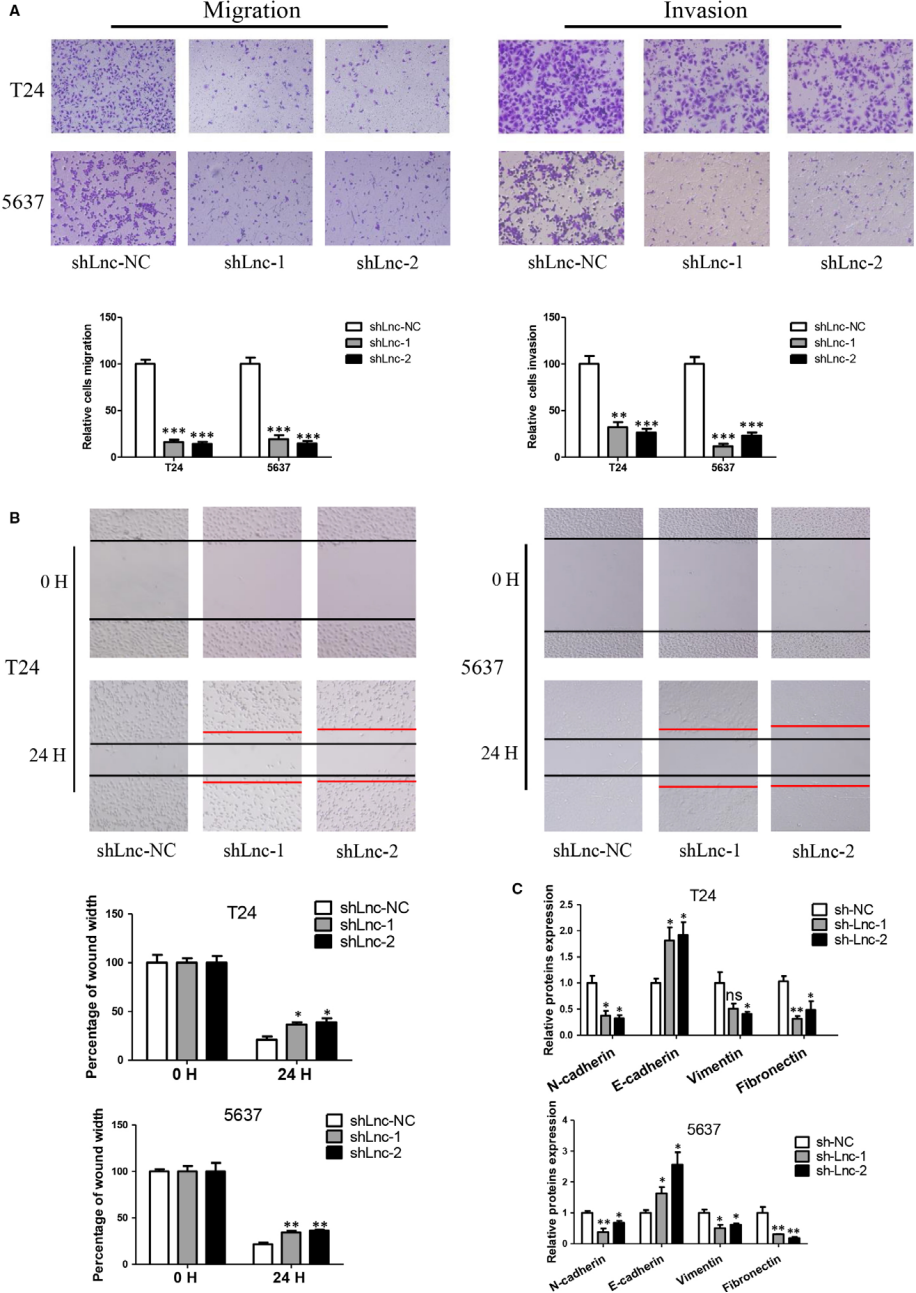
Knockdown of GAS6‐AS2 suppressed bladder cancer metastasic abilities via modification of EMT pathway. A, Knockdown of GAS6‐AS2 inhibited both migration and invasion abilities of T24 and 5637 cells. B, Knockdown of GAS6‐AS2 suppressed wound healing abilities of cells. C, Knockdown of GAS6‐AS2 altered EMT associated proteins. Data represent means ± SD of at least three independent experiments. *P < 0.05, **P < 0.01, ***P < 0.001

For metastasis was one of the most important independent risk factors that influence the prognosis of patients, we further explored the role of GAS6‐AS2 knockdown on metastatic abilities of bladder cancer cells. As shown in Figure 3A, both migration and invasion abilities were evaluated based on Transwell system. Our results showed the knockdown of GAS6‐AS2 could remarkable suppress the migration and invasion of T24 and 5637 cells. The wound healing assay further proved this pheromone (Figure 3B). Finally, the EMT markers were verified using Western blotting, and we proved knockdown of GAS6‐AS2 suppressed the EMT process (Figure 3C). In conclusion, we demonstrated that knockdown of GAS6‐AS2 inhibited metastatic abilities of bladder cancer cells via suppressing EMT pathways.

### 3.4 Overexpression of GAS6‐AS2 promoted proliferation and metastasis of bladder cancer cells

**Figure 4:**
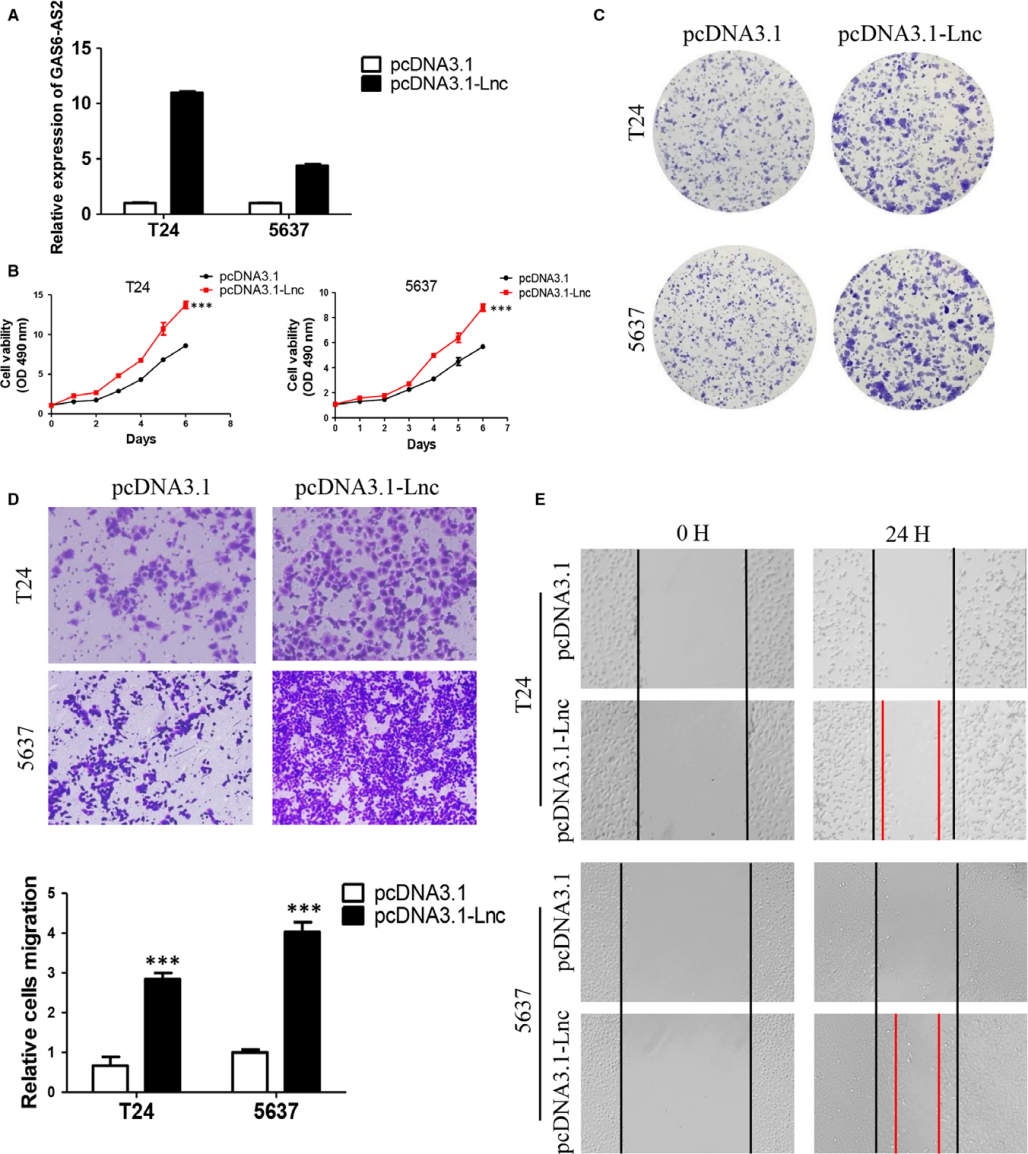
Overexpression of GAS6‐AS2 promoted proliferation and metastasis of bladder cancer cells. A, T24 and 5637 cells were overexpressed. B, Overexpression of GAS6‐AS2 promoted proliferation of bladder cancer cells. C, Overexpression of GAS6‐AS2 promoted colony formation ability. D, Overexpression of GAS6‐AS2 promoted migration of cells. E, Overexpression of GAS6‐AS2 promoted wound healing ability of bladder cancer cells. Data represent means ± SD of at least three independent experiments. ***P < 0.001

To further evaluate the roles of GAS6‐AS2 in bladder cancer, cells stable expression of GAS6‐AS2 were established and verified (Figure 4A). As shown in Figure 4B and C, we found that overexpression of GAS6‐AS2 contributed to both proliferation and colony formation abilities. Moreover, overexpression of GAS6‐AS2 also promoted metastatic abilities based on transwell and wound healing assays (Figure 4D and E). Based on our results, we further demonstrated overexpression of GAS6‐AS2 promoted proliferation and metastasis of bladder cancer cells.

### 3.5 GAS6‐AS2 functions as a ceRNA via directly sponging of miR‐298

**Figure 5:**
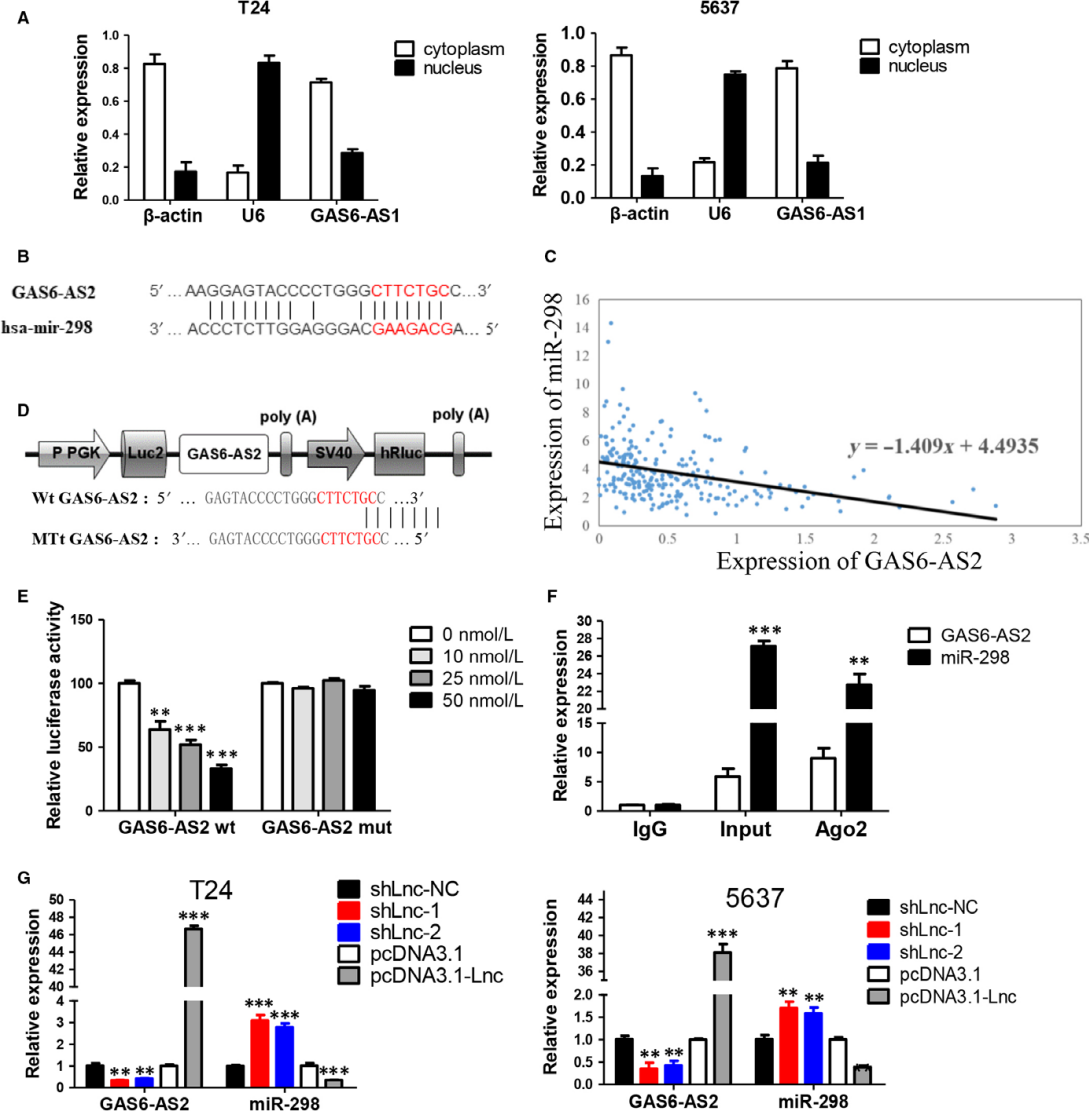
GAS6‐AS2 specifically sponged miR‐298. A, Detection of subcellular location of GAS6‐AS2 in both T24 and 5637 cell lines. B, The predicted targeting sequence of miR‐298 on the GAS6‐AS2. C, The correlation between GAS6‐AS2 and miR‐298 expression based on TCGA database. D, Construction of GAS6‐AS2 wild‐type and mutant type luciferase vectors. E, The wild‐type and mutant type of binding sites and luciferase reporter assay in HEK293T cells demonstrated combination between miR‐298 and GAS6‐AS2. F, Anti‐Ago2 RIP assay verified the combination between miR‐298 and GAS6‐AS2. G, Effect of GAS6‐AS2 knockdown or overexpression on miR‐298 expression. Data represent means ± SD of at least three independent experiments. **P < 0.01, ***P < 0.001

To further exploring the in‐depth mechanisms of GAS6‐AS2 involved in breast cancer progression, subcellular localization was firstly defined by nuclear mass separation experiment, and we found GAS6‐AS2 was mainly located in cytoplasm in both T24 and 5637 cell lines, which indicated that GAS6‐AS2 owned the potential ability of functioning as competitive endogenous RNA (ceRNA)^8^ (Figure 5A). Besides, bioinformatics prediction by RegRNA2.0^17^ and RNA22^16^ showed GAS6‐AS2 might directly bind with the seed sequence (2‐8 nucleotides) of miR‐298 (Figure 5B). Moreover, we further analysed the expression correlation between GAS6‐AS2 and miR‐298, which we found GAS6‐AS2 were negatively correlated with miR‐298 expression (Figure 5C).

To verify the direct combination of GAS6‐AS2 and miR‐298, fragments of wild‐type (wt) and mutated (mut) GAS6‐AS2 cDNA sequence containing the putative recognition site of miR‐298 was firstly cloned into pmir‐Glo (Figure 5D). Then dual reporter luciferase assay was performed in HEK293T cells cotransfected with both cloned vector and miR‐298 mimics based on gradient method. As shown in Figure 5E, with the increasing amount of transfected miR‐298 mimics, luciferase activities of pmir‐Glo‐GAS6‐AS2‐wt was significantly decreased while the luciferase activities in mut group showed no observable change. For Ago2 is a key component of RNA induced silencing complexes (RISC),^18^ immunoprecipitation was performed and both GAS6‐AS2 and miR‐298 could be pulled down by AGO2 antibody (Figure 5F), which indicated that GAS6‐AS2 could combine with AGO2 and miR‐298 in cell plasma and further function as a ceRNA. What's more, we found knockdown or overexpression of GAS6‐AS2 could negatively regulate the expression level of miR‐298 (Figure 5G). In conclusion, our results proved that GAS6‐AS2 functions as a ceRNA via directly sponging of miR‐298.

### 3.6 GAS6‐AS2 plays its functions via regulating GAS6‐AS2/miR‐298/CDK9 axis

**Figure 6:**
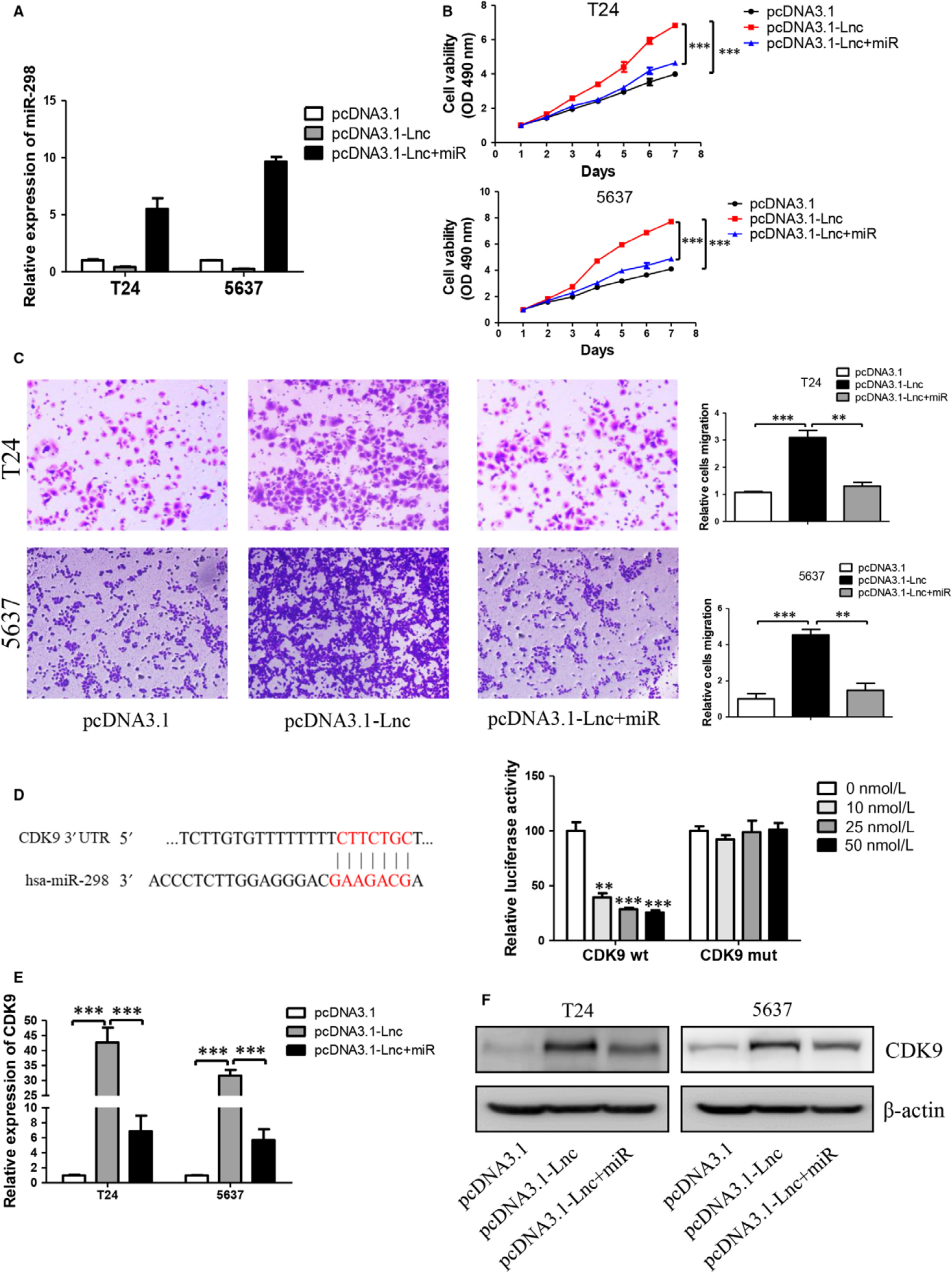
MiR‐298 antagonized effects of GAS6‐AS2 and directly targeting of CDK9. A, GAS6‐AS2 and/or miR‐298 were overexpressed in T24 and 5637 cells. B, MiR‐298 antagonized effect of GAS6‐AS2 on cell proliferation. C, MiR‐298 antagonized effect of GAS6‐AS2 on migration. D, The predicted targeting sequence of miR‐298 on the GAS6‐AS2. The wild‐type and mutant type of binding sites and luciferase reporter assay in HEK293T cells demonstrated combination between miR‐298 and CDK9. E, mRNA expression levels of CDK9 after GAS6‐AS2 and/or miR‐298 overexpression. F, Protein expression levels of CDK9 after GAS6‐AS2 and/or miR‐298 overexpression. Data represent means ± SD of at least three independent experiments. **P < 0.01, ***P < 0.001

For the verification of combination of GAS6‐AS2 and miR‐298, we further evaluated whether miR‐298 was a functional target of GAS6‐AS2, and explored its subsequently mechanisms. As shown in Figure 6A, T24 and 5637 cells overexpression GAS6‐AS2 cells and miR‐298 mimics were detected and the expression of miR‐298 were analysed. MTT assay and transwell assay of T24 and 5637 cells showed that miR‐298 mimics could antagonize effects of GAS6‐AS2 overexpression Figure 6B and C, which demonstrated miR‐298 was a functional target of GAS6‐AS2.

Next, we further explored mechanisms of GAS6‐AS2/miR‐298 axis and we found that CDK9 might be a target of miR‐298 based on Targetscan (Figure 6D).^19^ Then dual reporter luciferase assay was performed and our results showed a CDK9 was a direct target of miR‐298 (Figure 6E). Finally, based on qRT‐PCR and Western blotting assays, we found that GAS6‐AS2 increased while miR‐298 decreased the expression of CDK9 in both mRNA and protein levels (Figure 6F and G). Further evaluating roles of CDK9 in GAS6‐AS2/miR‐298 axis, MTT and transwell assay were performed. We found knockdown of CDK9 antagonized both proliferation and metastatic abilities of bladder cancer cells (Figure S1), which indicated that CDK9 was an important effector in GAS6‐AS2/miR‐298 axis. In conclusion, our results demonstrated that GAS6‐AS2 could promote proliferation and metastasis of bladder cancer cells via regulating GAS6‐AS2/miR‐298/CDK9 axis.

### 3.7 GAS6‐AS2 overexpression contributes to proliferation and metastasis of bladder cancer cells in vivo

**Figure 7:**
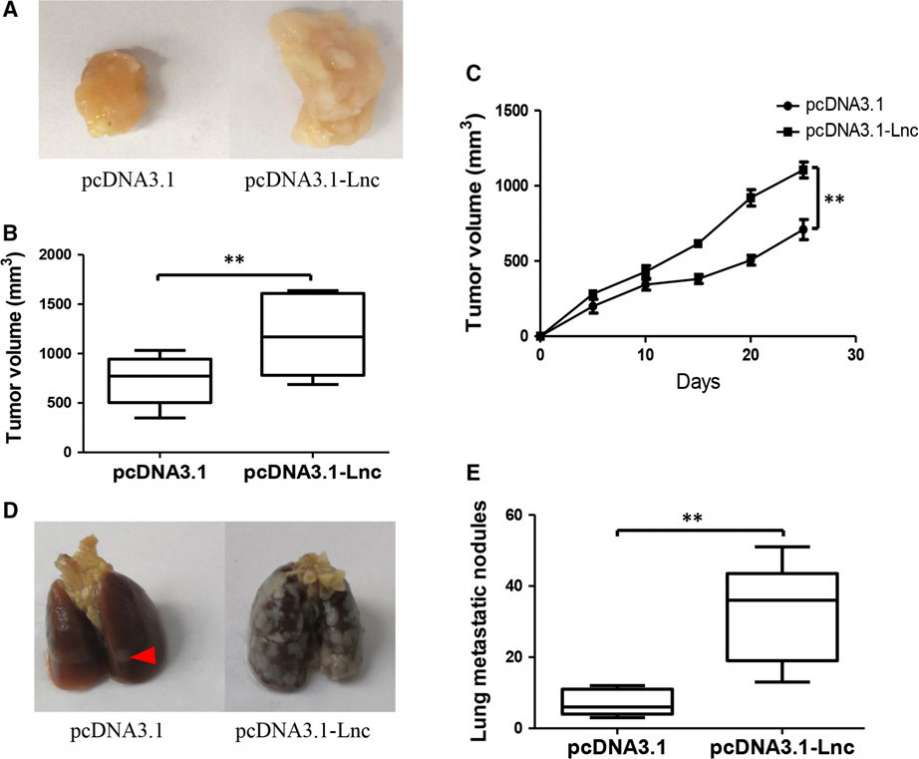
GAS6‐AS2 contributed to proliferation and metastasis of bladder cancer in vivo. A, Representative tumours in the two groups after 25 days of flank injection. B, The volume of tumours in the two groups. C, Growth curves of xenograft tumours after the injection of T24 cells. D, Representative lung metastasis in the two groups after 25 days of flank injection. E, The lung metastatic nodules in the two groups. Data represent means ± SD of at least three independent experiments. **P < 0.01

For the above results indicated that GAS6‐AS2 was a protooncogene in bladder cancer which promoted proliferation and metastasis of bladder cancer cells, we further evaluated its functions in vivo. As shown in Figure 7A‐C the tumour volume was significantly enlarged in GAS6‐AS2 overexpression group, which was similar to our in vitro results. For metastatic ability, we also found that the lung metastasis nodules were remarkably increased after GAS6‐AS2 overexpression (Figure 7D and E). In conclusion, our results further proved that GAS6‐AS2 overexpression contributes to proliferation and metastasis of bladder cancer cells in vivo.

## 4 DISCUSSION

The study of long noncoding RNAs in its function and mechanism is a rapidly expanding field with significant implications for understanding of disease. It have been reported that about 60 000 lncRNAs have been identified, which account for about 60% of the cellular transcriptome,^20^ and only no more than 4000 lncRNAs have been demonstrated aberrant expression and functional in cancers till now.^21^ More and more studies have confirmed that lncRNAs could regulate the malignant behaviours of tumour cells through its regulation of gene expression and functions.^4^ For instance, in bladder cancer, Hu et al reported that XIST promotes cell growth and metastasis through regulating miR‐139‐5p mediated Wnt/β‐catenin signalling pathway in bladder cancer.^22^ LncRNA UCA1 Promotes mitochondrial function of bladder cancer via the miR‐195/ARL2 signalling pathway.^23^ Based on previous studies, lncRNAs could function as biomarkers and play important roles in bladder cancer, while lncRNAs were far away from being understood. In this study, we firstly reported a novel lncRNAs termed GAS6‐AS2, promoted proliferation and metastasis of bladder cancer cells via GAS6‐AS2/miR‐298/CDK9 axis, which might serve as a potential biomarker for clinical diagnosis and treatment of bladder cancers.

After screening of aberrantly expressed lncRNAs between normal bladder tissues and bladder cancer tissues based on TCGA database, GAS6‐AS2 were selected for significantly overexpressed in cancer tissues. Then patients were further analysed based on clinical data, we found that GAS6‐AS2 was positively correlated with tumour stages, and GAS6‐AS2 overexpression predicted a poorer prognosis of bladder cancer patients. Moreover, we found that GAS6‐AS2 was overexpressed in bladder cancer cell lines compared with normal bladder cell lines, which further indicated potential roles of GAS6‐AS2 in bladder cancer. To verify our hypothesis, we first knockdowned GAS6‐AS2 expression using shRNAs, and we found that both shRNA vectors showed same effects to T24 and 5637 cells, in which the proliferation abilities were inhibited via induction of G1 phase arrest and the metastasic abilities were suppressed via regulation of EMT pathway (Figures 2 and 3). Moreover, we further overexpressed GAS6‐AS2 in both cell lines and in accordance with above results, overexpression of GAS6‐AS2 promoted proliferation and metastasis of bladder cancer cells (Figure 4).

As we have proved the important functions of GAS6‐AS2 on proliferation and metastasis of bladder cancer cells, which further identified that GAS6‐AS2 could function as a ceRNA via direct targeting of miR‐298. MiR‐298 has been identified as cancer‐suppressor gene in ovarian and breast cancer in which previous studies reported that miR‐298 inhibited malignant phenotypes of epithelial ovarian cancer and miR‐298 increased chemosensitivity of breast cancer cells to doxorubicin.^24^, ^25^ However, in gastric cancer cells, miR‐298 promoted cell proliferation and inhibited apoptosis and served as a oncogenic gene,^26^ which were inconformity to results of breast cancer and epithelial ovarian cancer cells. In this study, we primarily demonstrated that miR‐298 served as functional target of GAS6‐AS2 which could inhibit malignant behaviours of bladder cancer cells (Figure 5). Knockdown of GAS6‐AS2 decreased while overexpression increased the expression of miR‐298, indicating that combination of miR‐298 with GAS6‐AS2 was involved in RNA induced silencing complexes (RISC).^18^ Further exploring the functions and mechanisms of GAS6‐AS2/miR‐298 axis, we found that miR‐298 mimics antagonized GAS6‐AS2 overexpression and miR‐298 further interacted and suppressed both mRNA and protein levels of CDK9 (Figure 6). CDK9 has been reported to play an important role in G1 phase cell cycle arrest^27^ and metastasis^28^ of cancers, and we further performed experiments to validate its functions. We found knockdown of CDK9 antagonized GAS6‐AS2 functions, which could explain the functions and mechanisms of GAS6‐AS2/miR‐298/CDK9 axis. Finally, we further performed in vivo experiments which found that GAS6‐AS2 overexpression promoted proliferation and metastasis of bladder cancer as in vitro results.

**Figure 8:**
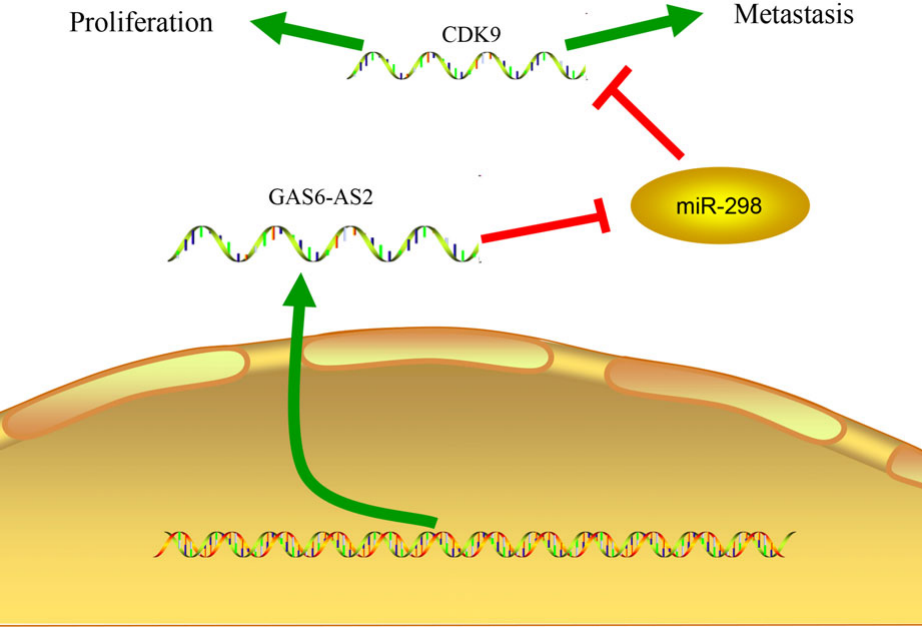
GAS6‐AS2 competitively combined with miR‐298 to increased CDK9 expression which contributes to bladder cancer proliferation and metastasis

In conclusion, we discovered a novel lncRNA termed GAS6‐AS2 that regulated the proliferation and metastasis via GAS6‐AS2/miR‐298/CDK9 axis (Figure 8). Our research broadens our insights into the underlying mechanisms in carcinogenesis and progression of bladder cancer, and provided a potential biomarker for clinical diagnosis and treatment of bladder cancer patient.

## CONFLICT OF INTEREST STATEMENT

The authors confirm that there are no conflicts of interest.
