# Supplementary material for: LncRNA GAS6‐AS2 promotes bladder cancer proliferation and metastasis via GAS6‐AS2/miR‐298/CDK9 axis
Source: J Cell Mol Med. 2018 Nov 5;23(2):865–76. doi: 10.1111/jcmm.13986 (PMC6349183; doi:10.1111/jcmm.13986)

**Figure S1**

**Knockdown of CDK9 antagonized effects of GAS6-AS2 on proliferation and metastasis.** (A) Expression of CDK9 after GAS6-AS2 overexpression and CDK9 inhibition. (B) CDK9 knockdown antagonized effect of GAS6-AS2 on cell proliferation. (C) CDK9 knockdown antagonized effect of GAS6-AS2 on migration. Data represent means ± S.D. of at least three independent experiments. *P < 0.05, **P < 0.01, ***P < 0.001.


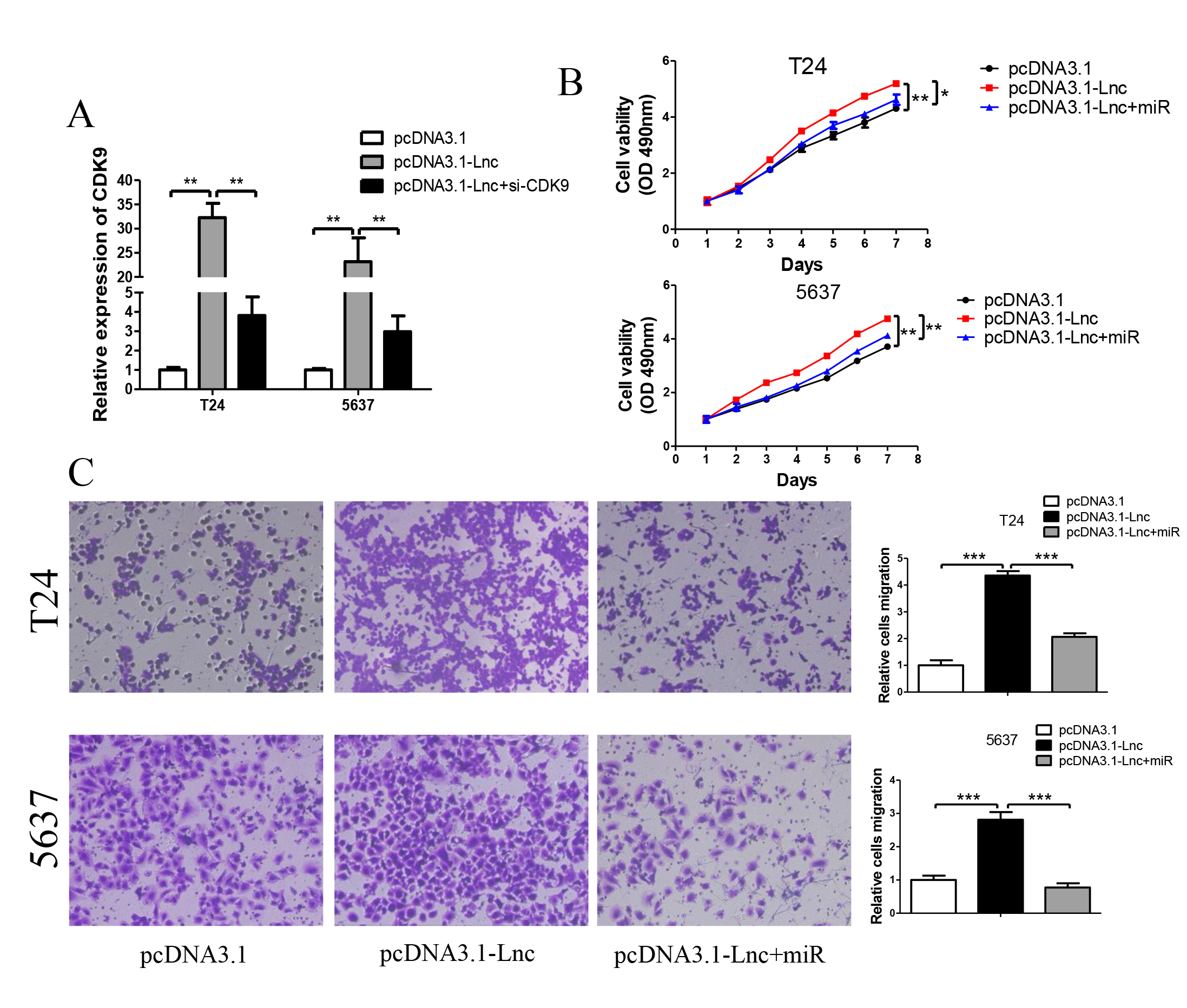

Supplement: Supplementary file 1 [file JCMM-23-865-s001.doc]
